# Kallmann Syndrome: Mutations in the Genes Encoding Prokineticin-2 and Prokineticin Receptor-2 

**DOI:** 10.1371/journal.pgen.0020175

**Published:** 2006-10-20

**Authors:** Catherine Dodé, Luis Teixeira, Jacqueline Levilliers, Corinne Fouveaut, Philippe Bouchard, Marie-Laure Kottler, James Lespinasse, Anne Lienhardt-Roussie, Michèle Mathieu, Alexandre Moerman, Graeme Morgan, Arnaud Murat, Jean-Edmont Toublanc, Slawomir Wolczynski, Marc Delpech, Christine Petit, Jacques Young, Jean-Pierre Hardelin

**Affiliations:** 1 Institut Cochin, INSERM U567, Université René Descartes, Paris, France; 2 Unité de Génétique des Déficits Sensoriels, INSERM U587, Institut Pasteur, Paris, France; 3 Laboratoire de Biochimie Génétique, Hôpital Cochin, Paris, France; 4 Service d'Endocrinologie, Hôpital Saint-Antoine, Paris, France; 5 Service de Génétique, Centre Hospitalier, Caen, France; 6 Laboratoire de Génétique Chromosomique, Centre Hospitalier, Chambéry, France; 7 Service d'Endocrinologie Pédiatrique, CHU Dupuytren, Limoges, France; 8 Département de Pédiatrie, Centre Hospitalier, Amiens, France; 9 Service de Génétique, Hôpital Jeanne de Flandres, Lille, France; 10 Sydney IVF, Sydney, Australia; 11 Clinique d'Endocrinologie, Maladies Métaboliques et Nutrition, Hôtel-Dieu, Nantes, France; 12 Service d'Endocrinologie Pédiatrique, Hôpital Saint-Vincent de Paul, Paris, France; 13 Department of Reproduction and Gynecological Endocrinology, Bialystok, Poland; 14 Service d'Endocrinologie, Hôpital de Bicêtre, Le Kremlin-Bicêtre, France; Johns Hopkins University School of Medicine, United States of America

## Abstract

Kallmann syndrome combines anosmia, related to defective olfactory bulb morphogenesis, and hypogonadism due to gonadotropin-releasing hormone deficiency. Loss-of-function mutations in *KAL1* and *FGFR1* underlie the X chromosome-linked form and an autosomal dominant form of the disease, respectively. Mutations in these genes, however, only account for approximately 20% of all Kallmann syndrome cases. In a cohort of 192 patients we took a candidate gene strategy and identified ten and four different point mutations in the genes encoding the G protein-coupled prokineticin receptor-2 *(PROKR2)* and one of its ligands, prokineticin-2 *(PROK2),* respectively. The mutations in *PROK2* were detected in the heterozygous state, whereas *PROKR2* mutations were found in the heterozygous, homozygous, or compound heterozygous state. In addition, one of the patients heterozygous for a *PROKR2* mutation was also carrying a missense mutation in *KAL1,* thus indicating a possible digenic inheritance of the disease in this individual. These findings reveal that insufficient prokineticin-signaling through PROKR2 leads to abnormal development of the olfactory system and reproductive axis in man. They also shed new light on the complex genetic transmission of Kallmann syndrome.

## Synopsis

Kallmann syndrome is a developmental disease that affects both the hormonal reproductive axis and the sense of smell. In addition, various nonreproductive and nonolfactory anomalies are occasionally observed in a fraction of the patients. There is a developmental link between the reproductive and olfactory disorders: neuroendocrine cells producing the gonadotropin-releasing hormone that is deficient in the patients normally migrate from the nose to the forebrain along olfactory nerve fibers during embryonic life, and they most probably fail to do so in the patients. Affected individuals usually do not undergo spontaneous puberty. Hormone replacement therapy is the treatment to initiate virilization in males or breast development in females, and later, to develop fertility in both sexes. This is a hereditary disease with complex genetic transmission. Mutations in either of two different genes, *KAL1* and *FGFR1,* have been found in approximately 20% of the affected individuals. The authors report on the identification (in a further 10% of patients) of various mutations in the prokineticin receptor-2 or prokineticin-2 genes, encoding a cell surface receptor and one of its ligands, respectively. Notably, some of the mutations were also detected in clinically unaffected individuals. This clearly indicates that additional, still unknown genetic or non-genetic factors are involved in disease production.

## Introduction

Kallmann syndrome (KS) combines hypogonadotropic hypogonadism and anosmia or hyposmia, i.e., a deficiency of the sense of smell [1]. Anosmia/hyposmia is related to the absence or hypoplasia of the olfactory bulbs and tracts [2]. Hypogonadism is due to deficiency in gonadotropin-releasing hormone [3] and probably results from a failure of embryonic migration of gonadotropin-releasing hormone-synthesizing neurons [4]. These cells normally migrate from the olfactory epithelium to the forebrain along the olfactory nerve pathway [5]. In some KS patients other developmental anomalies can be present, which include renal agenesis, cleft lip and/or palate, selective tooth agenesis, and bimanual synkinesis [6].

This is a genetically heterogeneous disease, which affects 1:8000 males and approximately five times less females. Two different genes have so far been identified. Loss-of-function mutations in *KAL1* (NCBI GeneID: 3730) [7–9] and *FGFR1* (NCBI GeneID: 2260) [10] account for the X-chromosome linked form and an autosomal dominant form of the disease, respectively. *KAL1* encodes anosmin-1, a locally restricted glycoprotein of embryonic extracellular matrices [11], which is likely to be involved in fibroblast growth factor-signaling [6,12]. Nearly 80% of the KS patients, however, do not carry a mutation in either of these genes [6].

Because the common infertility in affected individuals and, most importantly, the incomplete penetrance of the disease impede linkage analysis, the positional cloning strategies that have been taken to find causative genes were based on the analysis of rare KS individuals who carry chromosomal rearrangements detectable by cytogenetics techniques [7,8,10]. Here, we used a direct candidate gene approach and identified two novel genes underlying the disease.

## Results/Discussion

**Figure 1:**
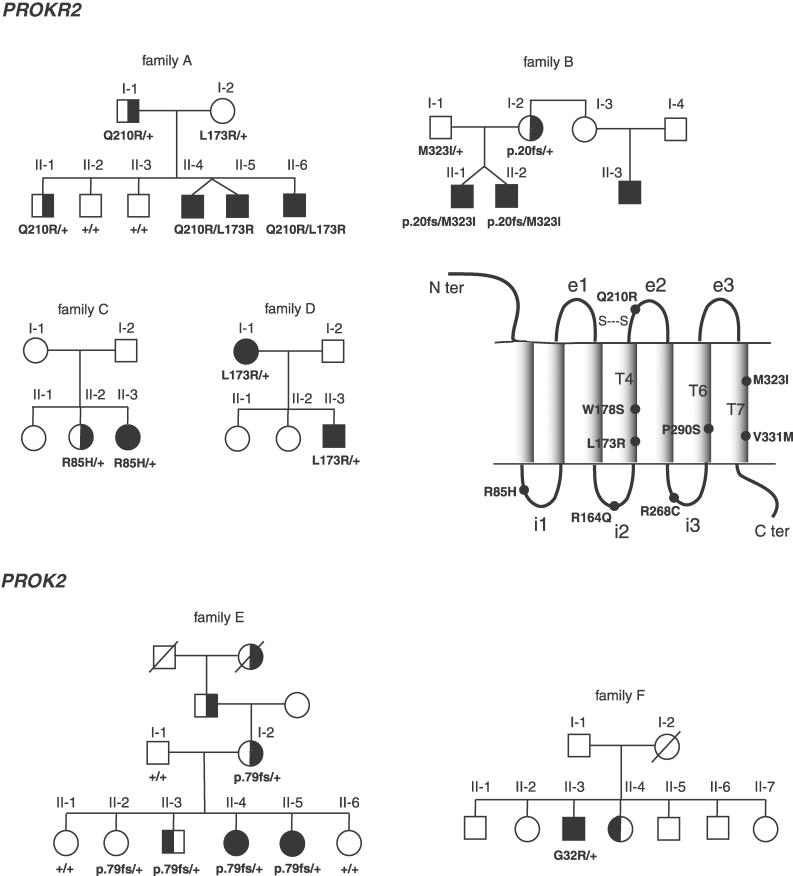
Segregation of Kallmann Syndrome and the *PROKR2* or *PROK2* Mutations in Affected Families Filled symbols denote clinically affected individuals with both hypogonadism and anosmia (or hyposmia). Half-filled symbols denote individuals with either anosmia only (right black part) or hypogonadism only (left black part). Genotypes, if available, are indicated below. The symbol + denotes normal allele, and fs stands for frameshift mutation. In several pedigrees the mutation is associated with varying phenotypes. Notably, in family A the disease apparently segregates according to a semi-dominant mode of transmission. The schematic representation of PROKR2 shows the locations of the nine missense mutations found in familial and non-familial KS cases, with respect to the putative N-terminal (N ter), C-terminal (C ter), extracellular loop (e1-e3), intracellular loop (i1-i3), and transmembrane (T1–T7) domains [13] of this G protein-coupled receptor.

We first considered *GPR73L1/PROKR2* (NCBI GeneID: 128674), encoding the prokineticin receptor-2 (PROKR2) [13–15], a most relevant candidate because olfactory bulbs do not develop normally in mutant mice lacking this G protein-coupled transmembrane receptor, and these mice also have a severe atrophia of the reproductive system related to the absence of gonadotropin-releasing hormone-synthesizing neurons in the hypothalamus [16]. We thus sequenced the two coding exons of *PROKR2* and flanking splice sites in 192 unrelated individuals (144 males and 48 females) affected by KS**,** including 38 familial cases. Ten different mutations (one frameshift and nine missense mutations) were detected in 14 patients (four familial and ten apparently sporadic cases) in the heterozygous (ten cases), homozygous (two cases), or compound heterozygous (two cases) state (Figure S1, Table 1, and Figure 1). Conservation of the mutated amino acid residues in bovine, murine, and rat orthologous sequences (Figure S2) argues in favor of a deleterious effect for all the missense mutations. However, two of these mutations, p.R268C and p.V331M, as well as a mutation (c.253C>T, p.R85C) affecting the same residue as the p.R85H mutation found in two KS cases and another missense mutation (c.1004C>G, p.T335M) not found in the cohort of KS patients, were detected, once each, in 500 alleles from ethnically matched (Caucasian) control individuals. No other nonsynonymous variant was found in the controls. In the absence of functional testing, one cannot be sure that each missense mutation found in KS individuals is causative of the disease. Nevertheless, together with the KS-like phenotype of *Prokr2* knockout mice, the fact that the overall proportion of *PROKR2* alleles carrying nonsynonymous mutations is significantly higher in KS patients (18 out of 384 alleles) than in controls (four out of 500 alleles; chi-square value = 13.5, *p* < 0.001) strongly argues in favor of the involvement of the gene in KS.

**Table 1:**
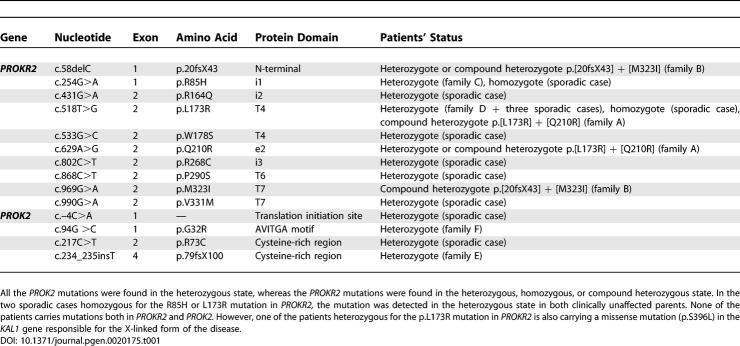
*PROKR2* and *PROK2* Mutations in Kallmann Syndrome

Prokineticin-2 (PROK2) [17] is the main ligand of PROKR2. We considered the possibility that mutations in *PROK2* (NCBI GeneID: 60675), account for some KS cases, especially since mutant mice defective in Prok2 have a marked reduction in the size of olfactory bulbs and a loss of their normal architecture [18]. We sequenced the four *PROK2* coding exons (including the alternative exon 3 [19]) and flanking splice sites in the same cohort of patients and found four different point mutations (two missense mutations, one frameshift mutation, and one single nucleotide substitution in the translation initiation sequence [20]), all in the heterozygous state, in two familial and two apparently sporadic cases (Figure S1, Table 1, and Figure 1). These mutations were not detected, or any other sequence variant, in 500 alleles from ethnically matched (Caucasian) control individuals. The p.G32R mutation affects the glycine residue of the N-terminal hexapeptide AVITGA (see Figure S2). This motif, which is conserved among prokineticins from mammalian and non-mammalian species, is critical for the bioactivities of these proteins [21]. The p.R73C mutation introduces a cysteine residue, which is expected to disrupt the formation of the disulfide bonds of the protein [19,21].

Considering both the phenotypes of the Prokr2- or Prok2-deficient mice and the likely deleterious effect of the human frameshift mutations on protein synthesis, the various *PROKR2* and *PROK2* missense mutations underlying KS are predicted to be loss-of-function mutations too. Since the same developmental disease, namely KS, apparently results from insufficient signaling either through FGFR1 or through PROKR2, the two signaling pathways are expected to interfere at some level, possibly the activation of the mitogen-activated protein kinase pathway [13,14,22]. In addition, the *KAL1* gene product anosmin-1, which has been reported to enhance fibroblast growth-factor-signaling through FGFR1 [12], could also play a role in prokineticin-signaling through PROKR2. Indeed, both anosmin-1 and PROK2 have binding affinities for heparan sulfate glycosaminoglycans, which are also well known co-receptors in fibroblast growth-factor-signaling [23–25]. Notably, only homozygous mouse mutants lacking Prokr2, Prok2, or Fgfr1 show abnormal olfactory bulbs [16,18,26], whereas it seems that heterozygous mutations in any of the orthologous genes can cause KS in humans ([10] and this study). A unifying explanation could be that the local amount of anosmin-1 exerts a critical dosage effect both on FGFR1 and PROKR2 receptor activation, hence a higher dose of anosmin-1 in the mouse (the murine *Kal1* is expected to be pseudoautosomal [27,28], whereas the human *KAL1* is X chromosome-linked) could protect heterozygous mouse mutants from the developmental failure.

It is noteworthy that KS patients who carry mutations in *PROKR2* or *PROK2* have variable degrees of olfactory and reproductive dysfunction (see Figure 1). In addition, they do not seem to have any of the occasional clinical anomalies that have been reported in the previously characterized genetic forms of the disease, namely bimanual synkinesis, renal agenesis, dental agenesis, and cleft lip or palate. However, the KS patient carrying the p.R73C mutation in *PROK2* suffers from a severe sleep disorder and marked obesity, which might be related to the known circadian function of prokineticin-2 and its potential roles in sleep-wake regulation and ingestive behavior [29–31].

In human monogenic disorders, genuine dominance, where heterozygotes and homozygotes have the same phenotype, is unusual. In particular, most dominant developmental diseases are far more severe in the homozygous state. Therefore, the finding of both heterozygous and homozygous (or compound heterozygous) KS patients for a given mutation in *PROKR2* (e.g., p.R85H or p.L173R, see Table 1) is quite remarkable and raises the question of a possible digenic mode of inheritance in heterozygous patients. In our cohort, none of the patients carrying a mutation in *PROKR2* was carrying a mutation in *PROK2* too. We did not find a mutation in *FGFR1* in any of the individuals carrying a mutation in *PROKR2* or *PROK2,* either. However, one of the patients heterozygous for the p.L173R mutation in *PROKR2* (sporadic case) also carried a previously undescribed missense mutation, p.S396L, in *KAL1* exon 8 (Figure S3), which was not detected in 500 alleles from control individuals. According to the predicted structure of anosmin-1 [32,33], the mutation modifies the first amino acid residue of the linker between the second and third fibronectin-like type III repeats [6], a residue that is conserved among orthologous proteins from vertebrates and invertebrates (Figure S3). This mutation thus can be regarded as causative of the disease. Likewise, the *PROKR2* mutation carried by this patient, L173R, which was found in six unrelated KS individuals and none of the controls, is most likely pathogenic too. To date, this is the only case of possible digenic inheritance reported in KS. It is quite possible, however, that other patients heterozygous for mutations in *PROKR2* or *PROK2* also carry a mutation in another, still unknown KS gene. Indeed, mutations in *KAL1, FGFR1, PROKR2,* and *PROK2* together account for approximately 30% of all the KS cases in our large series of patients, which indicates that still other genes underlying the disease remain undiscovered.

## Methods

Written informed consent was obtained from all the individuals who participated in the study. Genomic DNA was obtained from peripheral blood samples or lymphoblastoid cell lines by using a standard phenol chloroform extraction procedure. The genomic DNA from 250 unrelated Caucasian individuals was used as a control (control individuals were not examined to look for Kallmann syndrome features). We used the ENSEMBL (http://www.ensembl.org) and UCSC (http://www.genome.ucsc.edu) genome databases to find the exon-intron structure and single nucleotide polymorphisms of *PROK2* and *PROKR2.* We designed PCR primer sets for amplification of exons and exon-intron boundaries using PRIMER 3 software (http://frodo.wi.mit.edu/cgi-bin/primer3/primer3_www.cgi). Sequences of the primers used to amplify and sequence the *PROK2* four-coding exons and *PROKR2* two-coding exons in the patients and controls are listed below. The primers used for the *KAL1* and *FGFR1* coding exons have been reported [9,10]. All identified mutations were confirmed on a second PCR product. The alleles from control individuals were analyzed either by direct sequencing of the PCR products (*PROKR2* exons) or by using a denaturing high-performance liquid chromatography standard procedure followed by sequencing of abnormal products (*PROK2* exons, *KAL1* exon 8).

*PROK2* primers:
PROK2_1F: 5′-GGCGGGGCTAGCCTTTAT-3′
PROK2_1R: 5′-CCTCTAGCCTGCCCTTCAG-3′
PROK2_2F: 5′-CCCACTTTCGAAAAATGAGAA-3′
PROK2_2R: 5′-TGTTTGTCGAGCACGTTACC-3′
PROK2_3F: 5′-GGCTTGGCTGTATCTTGCTC-3′
PROK2_3R: 5′-TGGGGCTGAACTGATAGGAC-3′
PROK2_4F: 5′-GGGTAGTTAACGCTCAGTAAACA-3′
PROK2_4R: 5′-GAGCATTTCTTTCTGGCACA-3′
*PROKR2* primers:
PROKR2_1F: 5′-GGCTCACTGACCCTGAAAGA-3′
PROKR2_1R: 5′-TGTCAGCCTGTCAGAGCCTA-3′
PROKR2_2F: 5′-GGATTCACTGTGCCACTGC-3′
PROKR2_2R: 5′-CCATGCAGCCTATGAACTTG-3′

## Supporting Information

Figure S1 DNA Sequence Electrophoretograms for the Four *PROK2* Mutations and Ten *PROKR2* Mutations Found in Kallmann Syndrome Patients Normal sequences are shown on the top, mutated sequences at the bottom. Asterisks denote sequences from the non-coding DNA strand. Mutations are indicated by vertical arrows. All the mutations were found in the heterozygous or compound heterozygous state in Kallmann syndrome patients. In addition, two *PROKR2* mutations, namely c.254G>A (p.R85H) and c.518T>G (p.L173R), were also found in the homozygous state in one patient each.
(2.4 MB TIF)

Figure S2 Alignment of PROKR2 and PROK2 Amino Acid Sequences in Man, Cow, Mouse, and Rat (CLUSTALW) The missense mutations found in Kallmann syndrome patients are indicated by arrowheads. In the PROK2 sequence, the additional peptide encoded by exon 3 (alternative splicing) is underlined, and the N-terminal AVITGA motif that is critical for the bioactivity of the protein is highlighted in yellow.
(91 KB PDF)

Figure S3 DNA Sequence Electrophoretograms from the Kallmann Syndrome Patient Carrying Missense Mutations in *PROKR2* and *KAL1,* and Interspecies Comparison of the Amino Acid Sequence of KAL1 (Anosmin-1) around the Mutated Residue Control electrophoretograms are shown on the top. The mutations in *PROKR2* and *KAL1* are indicated by vertical arrows on the patient's electrophoretograms (bottom).
Alignment of the KAL1 amino acid sequences from man, cow, chicken, zebrafish (kal1.1 and kal1.2), *Caenorhabditis elegans,* and *Drosophila melanogaster* shows the conservation of the mutated residue (Ser396) in vertebrates and invertebrates (either serine or threonine), whereas most of the surrounding residues are more variable.
(943 KB TIF)

## Abbreviations

KS: Kallmann syndrome
*PROK2*: prokineticin-2
*PROKR2*: prokineticin receptor-2
