# Supplementary material for: Kallmann Syndrome: Mutations in the Genes Encoding Prokineticin-2 and Prokineticin Receptor-2
Source: PLoS Genet. 2006 Oct 20;2(10):e175. doi: 10.1371/journal.pgen.0020175 (PMC1617130; doi:10.1371/journal.pgen.0020175)
Supplement: Supplementary file 1 [file pgen.0020175.sg002.pdf]

PROKR2

```
                                                                                    R85H
                                                                                     ▼                                                 130
                                                                                                                                        |
PROKR2_human  MAAQNGNTSFTPNFNPPQDHASSLSFNFSYGDYDLPMDEDEDMTKTRTFFAAKIVIGIALAGIMLVCGIGNFVFIAALTRYKKLRNLTNLLIANLAISDFLVAIICCPFEMDYYVVRQLSWEHGHVLCAS
PROKR2_bovine MAAQNGNASFPANFSIPQEHASSLPFNFSYDDYDLPLDEDEDMTKTQTFFAAKIVIGVALVGIMLTCGIGNFVFITALTRYKKLRNLTNLLIANLAISDFLVAIICCPFEMDYYVVHQLSWEHGHVLCAC
PROKR2_mouse  MGPQNRNTSFAPDLNPPQDHVS---LNYSYGDYDLPLGEDEDVTKTQTFFAAKIVIGVALAGIMLVCGIGNFVFIAALARYKKLRNLTNLLIANLAISDFLVAIVCCPFEMDYYVVRQLSWAHGHVLCAS
PROKR2_rat    MGDQNGNTSFAPDLNPPQDHVSLLPLNYSYGDYDIPLDDDEDVTKTQTFFAAKIVIGVALAGIMLVCGVGNFVFIAALARYKKLRNLTNLLIANLAISDFLVAIVCCPFEMDYYVVRQLSWEHGHVLCAS
              *. ** *:**..::. **:*.*    :*:**.***:*:.:***:***:**********:**.****.**:******:**:*************************:***********:*** ********.

                                         R164Q  L173R  W178S                             Q210R
                                           ▼       ▼     ▼                                  ▼                                          260
                                                                                                                                        |
PROKR2_human  VNYLRTVSLYVSTNALLAIAIDRYLAIVHPLKPRMNYQTASFLIALVWMVSILIAIPSAYFATETVLFIVKSQEKIFCGQIWPVDQQLYYKSYFLFIFGVEFVGPVVTMTLCYARISRELWFKAVPGFQT
PROKR2_bovine INYLRTVSLYVSTNALLAIAIDRYLAIVHPLKPRMNYQTASFLIALVWMVSILISIPSAYFTKETVLFIVKNQKKIFCGQVWPVDQQLYYKSYFLFVFGIEFLGPVFTMTLCYARISRELWFKAVPGFQT
PROKR2_mouse  VNYLRTVSLYVSTNALLAIAIDRYLAIVHPLKPRMNYQTASFLIALVWMVSILIAVPSAYFTTETILVIVKNQEKIFCGQIWSVDQQLYYKSYFLFVFGLEFVGPVVTMTLCYARISQELWFKAVPGFQT
PROKR2_rat    VNYLRTVSLYVSTNALLAIAIDRYLAIVHPLK-RMNYQTASFLIALVWMVSILIAIPSAYFTTETILVIVKNQEKLFCGQIWPVDQQLYYKSYFLFVFGLEFVGPVVTMTLCYARISQELWFKAVPGFQT
              :******************************** *********************::*****:.**:*.***.*:*:****:*.*************:**:**:***.*********:************

                     R268C                P290S                                   M323I    V331M
                       ▼                    ▼                                       ▼        ▼                                   380
                                                                                                                                  |
PROKR2_human  EQIRKRLRCRRKTVLVLMCILTAYVLCWAPFYGFTIVRDFFPTVFVKEKHYLTAFYVVECIAMSNSMINTVCFVTVKNNTMKYFKKMMLLHWRPSQRGSKSSADLDLRTNGVPTTEEVDCIRLK
PROKR2_bovine EQIRKRLRCRRKTVLVLMCILTAYVLCWAPFYGFTIVRDFFPTVFVKEKHYLTAFYVVECIAMSNSMINTVCFVTVKNSTMKYFKKMLLLHWRPSHHGSKSSADLDLKTSRLPATEEVDCIRLK
PROKR2_mouse  EQIRKRLRCRRKTVLLLMGILTAYVLCWAPFYGFTIVRDFFPTVVVKEKHYLTAFYVVECIAMSNSMINTICFVTVKNNTMKYFKKMLRLHWRPSHYGSKSSADLDLKTSGVPATEEVDCIRLK
PROKR2_rat    EQIRKRLRCRRKTVLLLMGILTAYVLCWAPFYGFTIVRDFFPTLVVKEKHYLTAFYVVECIAMSNSMINTICFVTVKNNTMKYFKKMLLLHWRPSHYGSKSSADLDLKTSGVPATEEVDCIRLK
              ***************:** ***********************:.************************:*******.********: ******: **********:*. :*:**********
```

PROK2

```
                                         G32R                                    R73C
                                          ▼                                        ▼                                     120
                                                                                                                           |
PROK2_human   MRSLCCAPLLLLLLLPPLLLTPRAGDAAVITGACDKDSQCGGGMCCAVSIWVKSIRICTPMGKLGDSCHPLTRKNNFGNGRQERRKRKRSKRKKEVPFFGRRMHHTCPCLPGLACLRTSFNRFICLAQK
PROK2_bovine  MRSSRCARLLLLLLLPPLLLTPPAGDAAVITGACDRDPQCGGGMCCAVSLWVKSIRICTPMGKVGDSCHPMTRKNHFGNGRQERRKRKR-RRKKKVPFLGRRMHHTCPCLPGLACSRTSFNRYTCLAQK
PROK2_mouse   MGDPRCAPLLLLLLLP-LLFTPPAGDAAVITGACDKDSQCGGGMCCAVSIWVKSIRICTPMGQVGDSCHPLTRKSHVANGRQERRAKRRKRKKEVPFWGRRMHHTCPCLPGLACLRTSFNRFICLARK
PROK2_rat     MEDPRCAPLLLLLLLP-LLLTPPAGDAAVITGACDKDSQCGGGMCCAVSIWVKSIRICTPMGQVGDSCHPLTRKSHVANGRQERRAKRRKRKKEVPFWGRRMHHTCPCLPGLACLRTSFNRFICLARK
              * .  ** ******** **:** ************:*.**********:************::******:***.:..*******: ** :***:*** **************** ******: ***:*
```
